# Two phenolic antioxidants in *Suoyang* enhance viability of •OH-damaged mesenchymal stem cells: comparison and mechanistic chemistry

**DOI:** 10.1186/s13065-017-0313-1

**Published:** 2017-08-25

**Authors:** Yulu Xie, Xican Li, Jieying Xu, Qian Jiang, Hong Xie, Jianfeng He, Dongfeng Chen

**Affiliations:** 1 School of Chinese Herbal Medicine, Guangzhou University of Chinese Medicine, Waihuan East Road No. 232, Guangzhou Higher Education Mega Center, Guangzhou, 510006 China; 2 Innovative Research & Development Laboratory of TCM, Guangzhou University of Chinese Medicine, Guangzhou, 510006 China; 3 School of Basic Medical Science, Guangzhou University of Chinese Medicine, Guangzhou, 510006 China; 4 The Research Center of Basic Integrative Medicine, Guangzhou University of Chinese Medicine, Guangzhou, 510006 China

**Keywords:** Phenolic antioxidants, *Suoyang*, Epicatechin, Luteolin-7-*O*-*β*-D-glucoside, Mesenchymal stem cells

## Abstract

**Background:**

*Suoyang* originates from a psammophyte named *Cynomorium songaricum* Rupr and has been known as a phenolic-antioxidant-enriched traditional Chinese herbal medicine. The present study attempted to investigate the protective effect of phenolic antioxidants in *Suoyang* towards •OH-mediated MSCs and then further discusses the chemical mechanisms.

**Methods:**

The lyophilized aqueous extract of *Suoyang* (LAS) was prepared and characterized using HPLC. Then, two phenolic antioxidant references, epicatechin and luteolin-7-*O*-*β*-D-glucoside, along with LAS, were investigated for their effects on the viability of •OH-treated MSCs using the 3-(4, 5-dimethylthiazol-2-yl)-2,5-diphenyl (MTT) assay. The comparison and mechanistic chemistry of epicatechin and luteolin-7-*O*-*β*-D-glucoside were further explored using various antioxidant assays, including PTIO•-scavenging, FRAP (ferric ion reducing antioxidant power), ABTS^+^•-scavenging, and DPPH•-scavenging. Their Fe^2+^-binding capacities were also compared using ultraviolet (UV) spectra.

**Results:**

The HPLC analysis indicated that there are 8 phenolic antioxidants in LAS, including epicatechin, luteolin-7-*O*-*β*-D-glucoside, gallic acid, protocatechuic acid, catechin, isoquercitrin, phlorizin, and naringenin. The MTT assay revealed that epicatechin could more effectively increase the survival of •OH-treated MSCs than luteolin-7-*O*-*β*-D-glucoside. Similarly, epicatechin exhibited higher antioxidant abilities than luteolin-7-*O*-*β*-D-glucoside in the DPPH•-scavenging, ABTS^+^•-scavenging, FRAP, and PTIO•-scavenging assays. In the Fe^2+^-binding assay, luteolin-7-*O*-*β*-D-glucoside gave a stronger UV peak at 600 nm, with *ε* = 2.62 × 10^6^ M^−1^ cm^−1^, while epicatechin produced two peaks at 450 nm (*ε* = 8.47 × 10^5^ M^−1^ cm^−1^) and 750 nm (*ε* = 9.68 × 10^5^ M^−1^ cm^−1^).

**Conclusion:**

As two reference antioxidants in *Suoyang*, epicatechin and luteolin-7-*O*-*β*-D-glucoside can enhance the viability of •OH-damaged MSCs. Such a beneficial effect may be from their antioxidant effects, including direct-antioxidant and indirect-antioxidant (i.e., Fe^2+^-binding) processes. In the direct-antioxidant process, proton (H^+^), one electron (***e***), or even hydrogen-atom (•H) transfer may occur to fulfill radical-scavenging (especially •OH-scavenging); in this aspect, epicatechin is superior to luteolin-7-*O*-*β*-D-glucoside due to the presence of more phenolic –OHs. The additional –OHs can also be responsible for the better cytoprotective effect. In terms of indirect-antioxidant potential, however, epicatechin is inferior to luteolin-7-*O*-*β*-D-glucoside due to the absence of a hydroxyl-keto moiety. These findings will provide new information about medicinal psammophytes for MSC transplantation.

**Electronic supplementary material:**

The online version of this article (doi:10.1186/s13065-017-0313-1) contains supplementary material, which is available to authorized users.

## Background

**Fig. 1:**
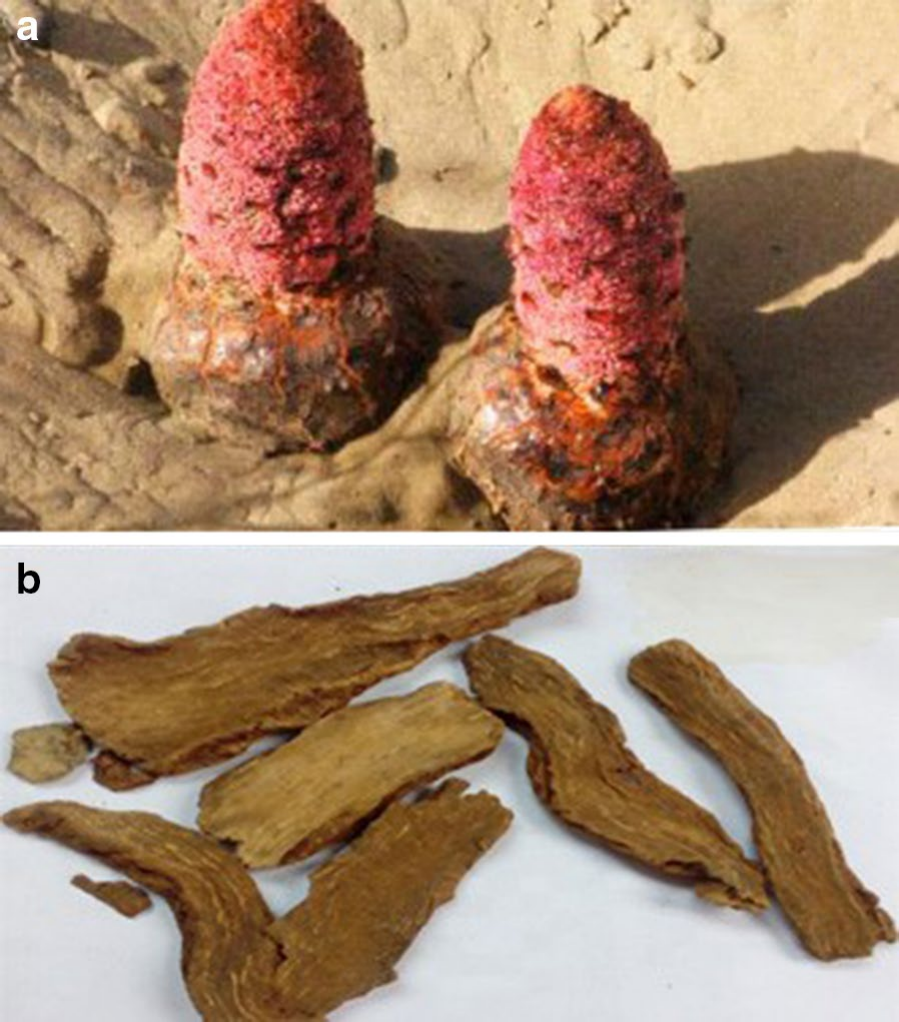
Photos of *Cynomorium songaricum* Rupr (**a**) and *Suoyang* (**b**)

A plant that grows in the desert or desert steppe is called a desert plant (or psammophyte). From the perspectives of free radical biology, desert plants may encounter a series of serious reactive oxygen species (ROS) damages from strong UV light, atmospheric ROS, great differences in temperature, and oxygen consumption for photosynthesis, since the ecological environment of the desert differs from that of land. Indeed, in such a hydropenia environment, the levels of ROS in plants will exceed the threshold value, and excessive ROS can oxidatively damage the proteins, nucleic acids, and enzymes, then lead to the death of plants [1]. Thus, some surviving desert plants have been suggested to have strong vital force and an effective antioxidant defense system against ROS-induced oxidative damage. The antioxidant defense systems can be classified into enzyme (including polypeptide) and non-enzyme systems. The non-enzyme defense system usually refers to phenolic antioxidants [2]. Hence, these surviving psammophytes are expected to be a library of bioactive components (especially efficient phenolic antioxidants). *Cynomorium songaricum* Rupr. (*C*. *songaricum,* Fig. 1a), a typical psammophyte, is widely distributed in the desert or desert steppe in the north–west provinces of China, Central Asian, Iran, and Mongolia. Phytochemical studies have indicated that there are various chemical components in *C*. *songaricum,* including organic acids, flavonoids, triterpenoids, steroids, volatile oils, saccharides, glucosides, tannins, lignans, alkaloids, amino acids, and mineral salts [3, 4]. Among them, flavonoids, glucosides, tannins, and lignans can act as phenolic antioxidants because of the presence of phenolic –OH in their molecules.

In traditional Chinese medicine (TCM), the stem (excluding the flower) of *C*. *songaricum* is used as a traditional Chinese herbal medicine called *Suoyang* (Fig. 1b). The *Suoyang* aqueous decoction can be used for the treatment of impotence and spermatorrhea, soreness and weakness of the waist and knees, and constipation. These functions in TCM seem to parallel the plant’s strong vital force in the desert.

Owing to the characteristics of psammophytes, the enrichment of phenolic antioxidants, and the functions in TCM, *Suoyang* has now attracted interest of researchers in the field of mesenchymal stem cells (MSCs) [5]. MSCs are known as an important stem cell type for tissue regenerative engineering [6]. However, they are in dire need of efficient phenolic antioxidants to resist against ROS-mediated (especially •OH-mediated) cellular death in the expansion process, which has been the bottleneck of MSC transplantation in clinical applications [7].

As a typical and most harmful form of ROS, the •OH radical has only a 10^−9^ s half-life and is prone to accumulate via the Fenton reaction, which frequently occurs in cells. The Fenton reaction is indispensable for some cellular physiological processes [8]. The accumulated •OH radical can cause substantial oxidative damage to cells [9]. Hence, •OH-mediated damage has become the major form of ROS-mediated cellular death. The present study aimed to investigate the possible protective effect of phenolic antioxidants in *Suoyang* towards •OH-mediated MSCs based on MTT assay, and then to explain the mechanisms of the cytoprotective effect using PTIO•-scavenging, DPPH•/ABTS•^+^-scavenging, FRAP, and Fe^2+^-binding assays. These findings highlight some important information on phenolic antioxidants from medicinal psammophytes in MSC transplantation engineering for clinical applications.

## Experimental

### Plant and animals

*Suoyang* (Xinjiang) (LOT. YPA6E0003) was purchased from Caizhilin Pharmaceuticals Co., Ltd. (Guangzhou, China). Sprague–Dawley (SD) rats of 4 weeks of age were obtained from the Animal Center of Guangzhou University of Chinese Medicine.

### Chemicals

Luteolin-7-*O*-*β*-D-glucoside (CAS 68321-11-9, 98%), protocatechuic acid (CAS 99-50-3, 98%), catechin (CAS 154-23-4, 98%), epicatechin (CAS 18829-70-4, 98%), naringenin (CAS 480-41-1, 98%), isoquercitrin (CAS 482-35-9, 98%), and phlorizin (CAS 60-81-1, 98%) were purchased from Weikeqi Biological Technology Co., Ltd. (Chengdu, China). Gallic acid (CAS 149-91-7, 98%) was purchased from Shanghai Aladdin Chemistry Co., Ltd. (Shanghai, China); Dulbecco’s modified Eagle’s medium (DMEM) and fetal bovine serum (FBS) were purchased from Gibco, Inc. (Grand Island, NY, USA). CD44 was purchased from Wuhan Boster Co., Ltd. (Wuhan, China). PTIO• (2-phenyl-4,4,5,5-tetramethylimidazoline-3-oxide-1-oxyl) was purchased from TCI (Shanghai) Development Co., Ltd. DPPH• (1,1-diphenyl-2-picryl-hydrazl), neocuproine (2,9-dimethyl-1,10-phenanthroline), TPTZ (2,4,6-tris(2-pyridyl-s-triazine)), Trolox [(±)-6-hydroxyl-2,5,7,8-tetramethlychroman-2-carboxylic acid], and the Percoll system were obtained from Sigma-Aldrich Trading Co. (Shanghai, China); (NH_4_)_2_ABTS [2,2′-azino-bis(3-ethylbenzo-thiazoline-6-sulfonic acid diammonium salt)] was purchased from Amresco Chemical Co. (Solon, OH, USA). Methanol and water were HPLC grade. All other reagents were analytical grade.

### Preparation of the lyophilized aqueous extract of *Suoyang* (LAS)

**Fig. 2:**
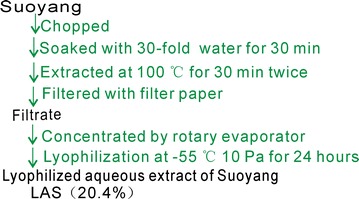
The flow chart of the preparation of the lyophilized aqueous extract of *Suoyang* (LAS)

The cut *Suoyang* was extracted with distilled water at 100 °C then freeze-dried to prepare the lyophilized aqueous extract of *Suoyang* (LAS). LAS with brownish red in appearance (Additional file 1) was stored at 4 °C for further analysis. The flow chart of preparation is shown in Fig. 2.

### HPLC characterization of LAS

HPLC analysis was performed using a Shimadzu LC-20A (Tokyo, Japan) equipped with an Agilent 5 TC-C18 250*4.6 mm column (Beijing, China). The mobile phase consisted of methanol (A) −0.3% and formic acid in water (C) (0.01 min, remain 5% A; 0–10 min, 5% A–10% A; 10–30 min, 10% A–40% A; 30–50 min, 40% A–50% A; 50–55 min, 50% A–5% A). The flow rate was 1.0 mL/min, the injection volume was 10 μL (400 mg/mL for LAS; 0.1 mg/mL for the standards), and absorption was measured at 280 nm [10]. In the study, phenolic components were identified by comparing their retention times, and the peak areas were employed to characterize the relative content of gallic acid, protocatechuic acid, catechin, epicatechin, luteolin-7-*O*-*β*-D-glucoside, isoquercitrin, phlorizin, and naringenin.

### Protective effect towards •OH-damaged MSCs (MTT assay)

MSCs were cultured according to the method described in our previous report [11]. In brief, bone marrow samples were accessed from the femurs and tibias of rats and diluted using low glucose DMEM containing 10% FBS. After gradient centrifugation at 900*g*/min for 30 min, the MSCs were prepared using a 1.073 g/mL Percoll system. The cells were then detached by treatment with 0.25% trypsin. The detached cells were passaged into culture flasks at a density of 1 × 10^−4^ cells/cm^2^. The homogeneity of the MSCs was evaluated at passage 3 based on their CD44 expression by flow cytometry. These cells were then used for the following experiments.

These MSCs were seeded into 96-well plates at a density of 4 × 10^3^ cells/well. After adherence for 24 h, the cells were classified into three groups, i.e., control group, model group, and samples group. The MSCs in the control group were incubated for 24 h in DMEM. The MSCs in the model group were injured for 25 min using FeCl_2_ (100 μM), followed by H_2_O_2_ (50 μM). The mixture of FeCl_2_ and H_2_O_2_ was removed, and the MSCs were incubated for 24 h in DMEM. The MSCs in the samples group were injured and incubated for 24 h in DMEM in the presence of various concentrations of samples. After incubation, 20 μL of MTT (5 mg/mL in PBS) was added to the cells, which were then incubated for 4 h. The culture medium was subsequently discarded and replaced with 150 μL of DMSO. The absorbance of each well was then measured at 490 nm using a Bio-Kinetics plate reader (PE-1420; Bio-Kinetics Corporation, Sioux Center, IA, USA). The serum medium was used for the control group, and each sample test was repeated in five independent wells.

### PTIO•-scavenging assay

The PTIO•-scavenging assay was conducted based on our method [12]. In brief, the test sample (*x* = 0–10 μL, 1 mg/mL) was added to (10 − *x*) μL of 95% ethanol, followed by 90 μL of an aqueous PTIO• solution (0.1 mM). The mixture was maintained at 37 °C for 2 h, and the absorbance was then measured at 560 nm using a microplate reader (Multiskan FC, Thermo Scientific, Shanghai, China). The PTIO• inhibition percentage was calculated as:

$${Inhibition\, \% =\, }\frac{{\text{A}_{\text{0}} - A}}{{\text{A}_{\text{0}} }}\text{ } \times { 100\% }$$,

where A_0_ is the absorbance of the control without the sample, and A is the absorbance of the reaction mixture with the sample.

### DPPH•-scavenging and ABTS^+^•-scavenging assays

The DPPH•-scavenging and ABTS^+^•-scavenging assays were based on previous reports [13]. In the DPPH•-scavenging assay, 90 μL of an ethanolic solution of DPPH• (0.1 mM) was mixed with (10 − *x*) μL of an ethanolic or (*x* = 0–10 μL, 0.2 mg/mL) aqueous solution of the sample. The mixture was maintained at room temperature for 30 min, and the absorbance was then measured at 519 nm. In the ABTS^+^•-scavenging assay, the ABTS^+^• was produced by mixing 200 μL of (NH_4_)_2_ABTS (7.4 mM) with 200 μL of K_2_S_2_O_8_ (2.6 mM). After incubation in the dark for 12 h, the mixture was diluted with methanol (approximately 1:50) so that the absorbance at 734 nm was 0.30 ± 0.01. Then, the diluted ABTS^+^• solution (90 μL) was added to (10 − *x*) μL of an ethanolic or (*x* = 0–10 μL, 0.1 mg/mL) aqueous solution of the sample and then mixed thoroughly. After the reaction mixture stood for 6 min, the absorbance was measured at 734 nm using a spectrophotometer. The percentage of inhibition of DPPH•-scavenging or ABTS^+^•-scavenging was calculated using the formula described in “PTIO•-scavenging assay” section.

### Ferric reducing antioxidant power (FRAP) assay

The FRAP assay was adapted from Benzie and Strain [14]. Briefly, the FRAP reagent was prepared fresh by mixing 10 mM TPTZ, 20 mM FeCl_3_ and 0.3 M acetate buffer at 1:1:10 at pH 3.6. The test sample (*x* = 0–20 μL, 0.5 mg/mL) was added to (20 − *x*) μL of 95% ethanol, followed by 80 μL of FRAP reagent. The absorbance was measured at 593 nm after a 30-min incubation at ambient temperature, using distilled water as the blank. The relative reducing power of the sample compared with the maximum absorbance was calculated by the following formula:

$$Relative \, reducing \, effect{\text{ \% }} = \, \frac{{A - A_{{{\text{min}}} } }}{{A_{{{\text{max}}} } - A_{{{\text{min}}} } }} \, \times {\text{ 100\% ,}}$$

where A_min_ is the absorbance of the control without the sample, A is the absorbance of the reaction mixture with the sample, and A_max_ is the greatest absorbance of the reaction mixture with the sample.

### Ultraviolet (UV) spectral determination of Fe^2+^-binding

Ultraviolet (UV) spectra of Fe^2+^-binding were conducted according to a previously described method [15]. Briefly, a 100-μL test sample was added to 100 μL of an aqueous solution of FeCl_2_·4H_2_O (10 mg/mL). The total volume was adjusted to 200 μL, and the solution was then mixed vigorously. The resulting mixture was incubated at room temperature for 24 h. The product mixtures were then imaged using a smartphone (Samsung, Galaxy A7, China). Subsequently, the supernatant was collected, and a spectrum was obtained using a UV/Vis spectrophotometer (Jinhua 754 PC, Shanghai, China) from 200 to 1000 nm.

### Statistical analysis

The IC_50_ values were calculated by linear regression analysis. All linear regression analyses in this study were analyzed by the Origin 6.0 professional software. The determination of significant differences between the mean IC_50_ values of the sample and positive controls was performed using one-way analysis of variance (ANOVA) and a *T* test. The analysis was performed using SPSS software 13.0 (SPSS Inc., Chicago, IL) for windows. *P* < 0.05 was considered to be statistically significant.

## Results and discussion

As mentioned above, *Suoyang* is used as an aqueous decoction for clinical application in TCM. Thus, the present study first used distilled water (not alcohols or other organic solvents) to prepare its extract. To avoid destroying the relevant phenolics, the aqueous extract was then lyophilized at −55 °C under a vacuum condition (10 Pa) for 24 h. The yield of the lyophilized aqueous extract of *Suoyang* (LAS) was calculated as 20.4% (Fig. 2).

**Fig. 3:**
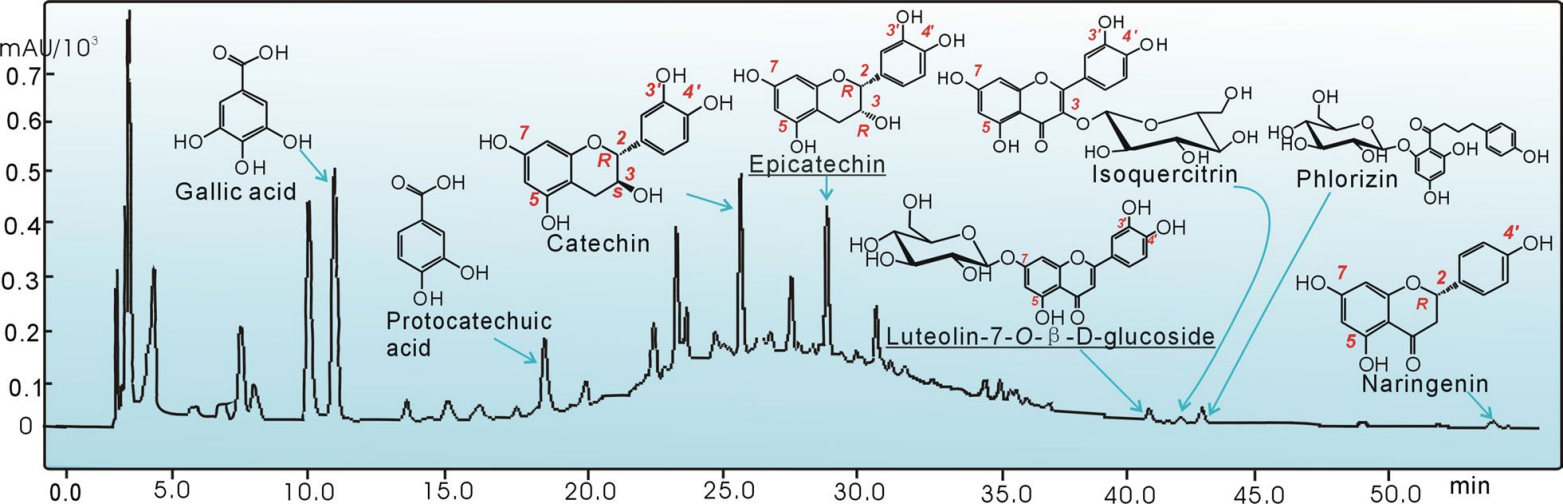
A typical HPLC profile of LAS (lyophilized aqueous extract of *Suoyang*)

The HPLC analysis indicated that LAS was comprised of at least 8 phenolic antioxidants, including gallic acid, protocatechuic acid, catechin, epicatechin, luteolin-7-*O*-*β*-D-glucoside, isoquercitrin, phlorizin, and naringenin (Fig. 3). These components have previously been demonstrated to exist in Suoyang [3, 4]. Among them, gallic acid [16, 17], protocatechuic acid [18], catechin [16, 19, 20], isoquercitrin [21], and naringenin [22] were mentioned in our previous studies, and phlorizin was recently observed to have proliferative potential of epidermal stem cells [23]. Thus, the present study focused on epicatechin and luteolin-7-*O*-*β*-D-glucoside. From the viewpoint of chemistry, there are exactly two representatives of phenolic antioxidants: epicatechin for tea-polyphenols and luteolin-7-*O*-*β*-D-glucoside for flavonoids.

As shown in Table 1, epicatechin and luteolin-7-*O*-*β*-D-glucoside dose-dependently increased their A_490 nm_ values in the MTT assay. These findings suggest that both epicatechin and luteolin-7-*O*-*β*-D-glucoside could protect MSCs from •OH-mediated damage. However, due to the low levels of epicatechin and luteolin-7-*O*-*β*-D-glucoside in LAS, LAS exhibited only slight activity in the MTT assay. Between epicatechin and luteolin-7-*O*-*β*-D-glucoside, the former had a stronger protective effect than the latter. This protective effect has been presumed to be attributed to the antioxidant action [24]. Our presumption is supported by the data listed in Table 2, where epicatechin usually presented lower IC_50_ values than luteolin-7-*O*-*β*-D-glucoside in the ABTS^+^• scavenging, DPPH• scavenging, PTIO• scavenging, and FRAP assays.

**Table 1:** The A _490nm_ of LAS, epicatechin, and luteolin-7-*O*-*β*-D-glucoside towards •OH-damaged MSCs in MTT assay

	LAS	Epicatechin	Luteolin-7-*O*-*β*-D-glucoside
Control	0.54 ± 0.18	0.54 ± 0.18	0.54 ± 0.18
Model	0.10 ± 0.00	0.11 ± 0.01	0.11 ± 0.01
10 μg	0.10 ± 0.01	0.12 ± 0.01	0.11 ± 0.01
30 μg	0.11 ± 0.02	0.17 ± 0.01	0.12 ± 0.00
50 μg	0.13 ± 0.02	0.22 ± 0.02	0.14 ± 0.02
100 μg	0.14 ± 0.00	0.24 ± 0.02	0.18 ± 0.01

Experiments were performed with 3 different batches of cells and each batch was tested in triplicate. The Fenton reagent (FeCl_2_ *plus* H_2_O_2_) was used to generate •OH radicals. These data represent the mean ± SD (*n* = 3). * *p* < 0.05 vs. model

*MTT* 3-(4,5-dimethylthiazol-2-yl)-2,5-diphenyltetrazolium bromide; *LAS* lyophilized aqueous extract of *Suoyang*

**Table 2:** The IC_50_ values of LAS, epicatechin, luteolin-7-O-β-D-glucoside in various antioxidant assays

Assays	LAS, μg/mg	Epicatechin, μg/mg (μM)	Luteolin-7-*O*-*β*-D-glucoside, μg/mg (μM)	Trolox, μg/mg (μM)
PTIO• scavenging	93.1 ± 0.6	82.6 ± 2.3^b^ (283.6 ± 7.2)^C^	205.9 ± 13.2^e^ (459.2 ± 2.9)^A^	55.5 ± 5.5^a^ (221.1 ± 22.0)^B^
FRAP	179.1 ± 3.7	11.3 ± 1.0^b^ (38.8 ± 3.4)^A^	15.6 ± 0.7^b^ (33.9 ± 2.5)^A^	13.4 ± 1.0^b^ (53.4 ± 4.1)^A^
DPPH• scavenging	188.5 ± 3.0	2.5 ± 1.8^a^ (8.6 ± 6.2)^A^	12.8 ± 0.4^b^ (28.6 ± 0.9)^A^	3.8 ± 0.5^b^ (15.0 ± 2.1)^A^
ABTS^+^• scavenging	70.5 ± 0.8	1.5 ± 0.8^a^ (5.3 ± 2.8)^A^	8.3 ± 0.4^b^ (18.6 ± 0.9)^A^	5.2 ± 0.4^b^ (20.8 ± 1.5)^A^

IC_50_ value is defined as the concentration of 50% effect percentage and expressed as mean ± SD (*n* = 3). The linear regression was analyzed by Origin 6.0 professional software. Means values with different superscripts (a, b or A, B) in the same row are significantly different (*p* < 0.05), while with same superscripts are not significantly different (*p* > 0.05)

*FRAP* ferric ion reducing antioxidant power, *Trolox* [(±)-6-hydroxyl-2, 5, 7, 8-tetramethlychromane-2-carboxylic acid] acts as the positive control, *LAS* lyophilized aqueous extract of *Suoyang*. The dose response curves were shown in Additional file 2

ABTS^+^•, DPPH•, and PTIO• radicals are similarly stable free radicals in vitro; thus, the reaction to scavenge these radicals is suggested to be a direct-antioxidant process. Nevertheless, their mechanisms are actually not identical. PTIO• scavenging is reported as a H^+^-transfer pathway [25]. The FRAP assay under pH 3.6 is regarded as an electron-transfer (ET) reaction [26]. The fact that LAS, epicatechin, and luteolin-7-*O*-*β*-D-glucoside have inhibitory effects on PTIO• and reductive effects on Fe^3+^ (Table 2; Additional file 2) implies that their direct-antioxidant action may include H^+^-transfer and ET pathways. The possibility of an ET pathway is partly supported by the results from the ABTS^+^• assay (Table 2; Additional file 2). In fact, the ABTS molecule was found to be oxidized via one-electron transfer to form the ABTS^+^• radical [27], and ABTS^+^• radical scavenging is used as an important model for one-electron transfer [28]. Of course, a possibility of H^+^ combining with an electron to simultaneously transfer should not be excluded; this is actually a HAT pathway. A series of reports mentioned that DPPH• scavenging, at least, includes a HAT pathway [29, 30], which partly supports the possibility of HAT in their antioxidant action. As shown in Table 2 and Additional file 2, LAS, epicatechin, and luteolin-7-*O*-*β*-D-glucoside exhibited good dose-dependency in the DPPH assay. Thus, their antioxidant mechanisms include a H^+^-transfer pathway and an ET pathway, which may include a HAT pathway. As stated by Musialik, they are nonexclusive mechanisms [30].

**Fig. 4:**
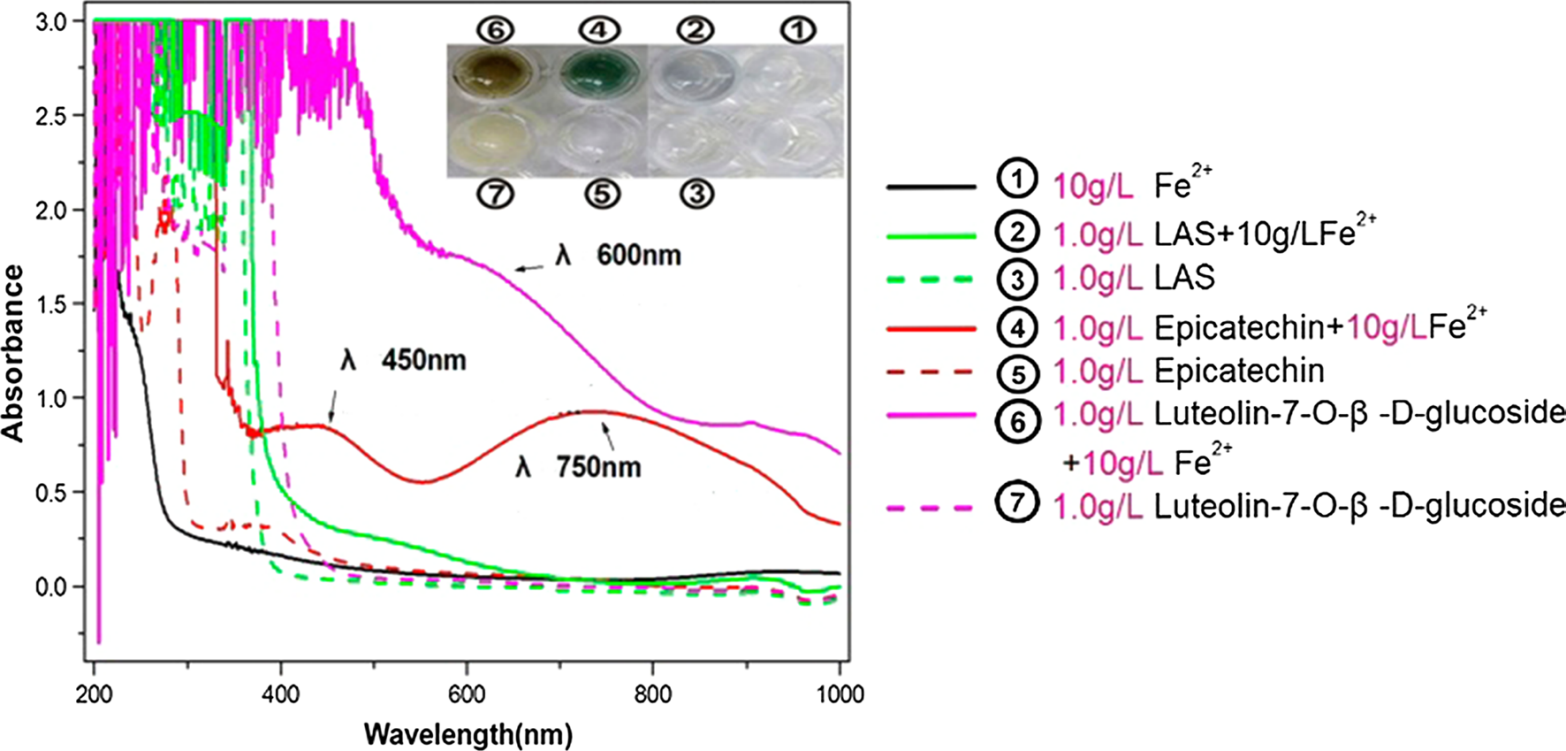
UV spectra of LAS, epicatechin, and luteolin-7-*O*-*β*-D-glucoside and their binding products with excess Fe^2+^ (the *inset figure* is the appearances of solutions; *LAS* lyophilized aqueous extract of *Suoyang*)

Unlike these stable radicals (e.g., PTIO•, DPPH•, and ABTS^+^•), the transient •OH radical usually relies on Fe^2+^ as a catalyst to be formed in cells. A typical example is the Fenton reaction (Fe^2+^ + H_2_O_2_ → Fe^3+^+ •OH + •OH^–^). Thus, attenuation of Fe^2+^ levels via a binding reaction is considered to be an indirect-antioxidant mechanism for scavenging •OH radicals. In fact, iron-binding by phenolic antioxidants have been focused on for their beneficial effects towards the diseases caused by oxidative stress [31]. In the present study, epicatechin and luteolin-7-*O*-*β*-D-glucoside exhibited higher Fe^2+^-binding abilities than LAS. As shown in Fig. 4, the epicatechin-Fe^2+^ complex gave two UV peaks at 450 and 750 nm, and the molar extinction coefficients were calculated as *ε* = 8.47 × 10^5^ M^−1^ cm^−1^, and 9.68 × 10^5^ M^−1^ cm^−1^, respectively; the luteolin-7-*O*-*β*-D-glucoside-Fe^2+^ complex yielded a strong shoulder-peak at 600 nm with *ε* = 2.62 × 10^6^ M^−1^ cm^−1^. This finding implies that Fe^2+^-binding may act as one pathway for the antioxidant action of LAS.

**Fig. 5:**
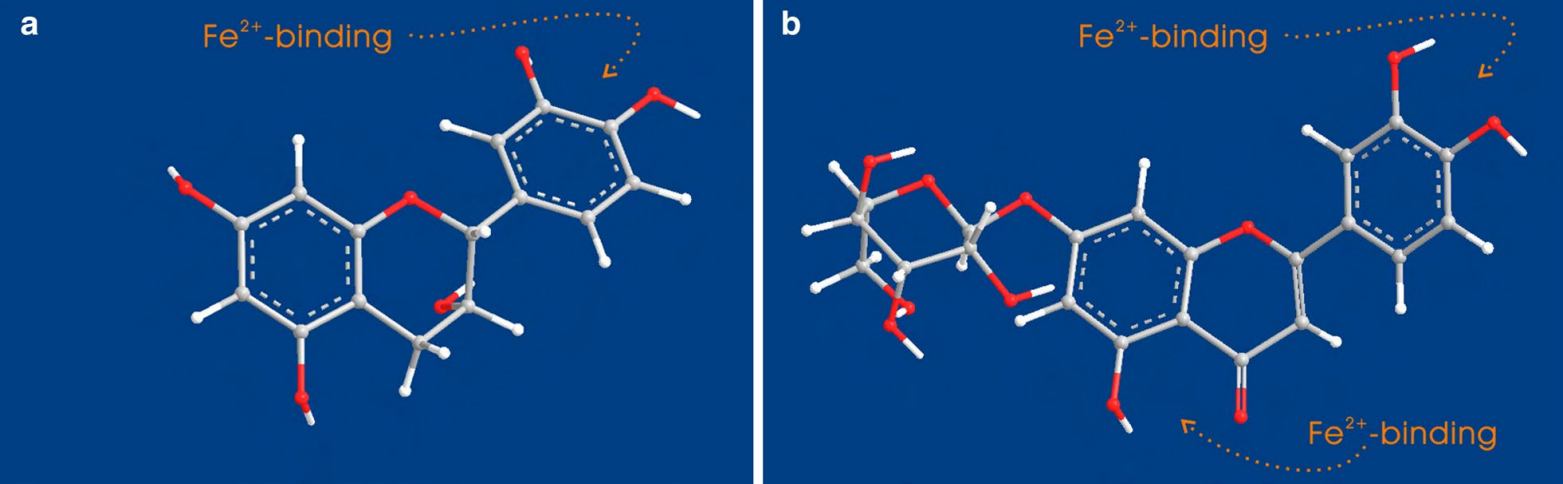
The preferential conformation-based, ball-stick models of epicatechin (**a**) and luteolin-7-*O*-*β*-D-glucoside (**b**)

**Fig. 6:**
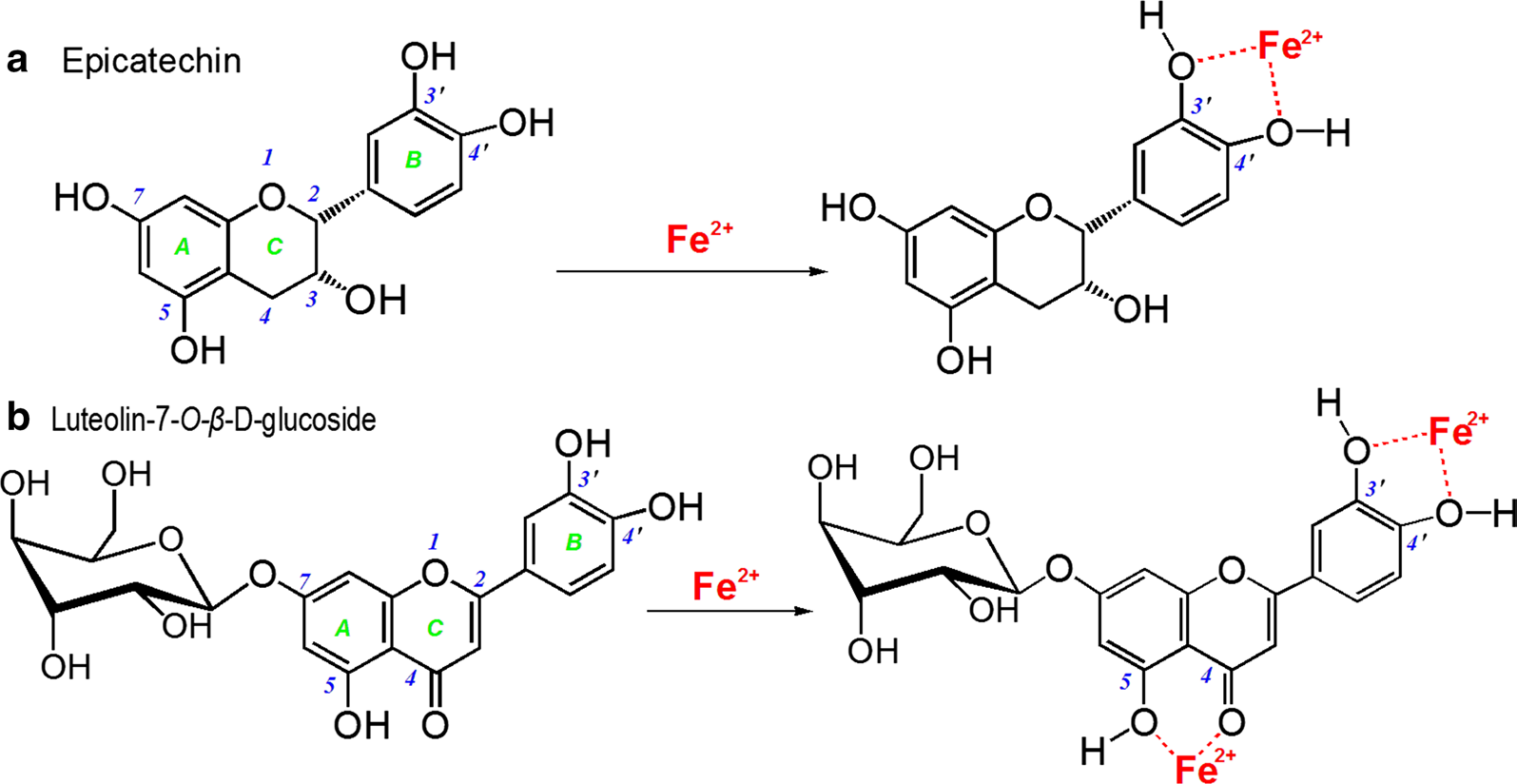
The proposed Fe^2+^-binding reactions with a catechol moiety in epicatechin (**a**) and with catechol and hydroxyl-keto moieties in luteolin-7-*O*-*β*-D-glucoside (**b**)

When comparing epicatechin and luteolin-7-*O*-*β*-D-glucoside, however, the former exhibited a lower ability than the latter in the Fe^2+^-binding reaction. As shown in Fig. 4, epicatechin resulted in a weaker UV peak and lighter color than luteolin-7-*O*-*β*-D-glucoside. As illustrated by the preferential conformation-based, ball-stick models, epicatechin has only one site for the metal-binding reaction, i.e., the 3′,4′-catechol moiety in B ring (Fig. 5a), while luteolin-7-*O*-*β*-D-glucoside possesses two binding sites, i.e., a 3′,4′-catechol moiety and a hydroxyl-keto moiety at the 4,5-position (Fig. 5b). The catechol moiety can bind Fe^2+^ to form a planar five-membered ring, while the hydroxyl-keto moiety can bind efficiently to a planar six-membered ring [21]. Thereby, luteolin-7-*O*-*β*-D-glucoside, with its two binding sites, produced higher Fe^2+^-binding peaks and a darker solution color than epicatechin with one binding site. Based on the above discussion and on previous literature [26, 32], we propose the Fe^2+^-binding reactions of epicatechin and luteolin-7-*O*-*β*-D-glucoside as follows (Fig. 6).

## Conclusion

There are at least 8 phenolic antioxidants in *Suoyang*, including epicatechin, luteolin-7-*O*-*β*-D-glucoside, gallic acid, protocatechuic acid, catechin, isoquercitrin, phlorizin, and naringenin. As two reference phenolic antioxidants in *Suoyang,* epicatechin and luteolin-7-*O*-*β*-D-glucoside have been proven to enhance the viability of •OH-damaged MSCs. Such a beneficial effect may result from their antioxidant effects, which may consist of direct-antioxidant processes and an indirect-antioxidant process (i.e., Fe^2+^-binding). The direct-antioxidant pathways include a H^+^-transfer and/or ET and possibly a HAT. In this aspect, epicatechin is superior to luteolin-7-*O*-*β*-D-glucoside; this finding can be attributed to the difference in the amount of phenolic –OHs, which is responsible for its more beneficial effect towards •OH-damaged MSCs. In terms of indirect-antioxidant potential, however, epicatechin is inferior to luteolin-7-*O*-*β*-D-glucoside, due to the absence of the hydroxyl-keto moiety.

## Additional files

**Additional file 1.** The appearance of the lyophilized aqueous extract of *Suoyang*.

**Additional file 2.** The dose response curves of PTIO, FRAP, DPPH, and ABTS assays.

## Abbreviations

ABTS: [2,2′-azino-bis(3-ethylbenzo-thiazoline-6-sulfonic acid diammonium salt)]
DMEM: Dulbecco’s modified Eagle’s medium
DPPH•: (1,1-diphenyl-2-picryl-hydrazl)
ET: electron transfer
FRAP: ferric reducing antioxidant power
HAT: hydrogen atom transfer
LAS: lyophilized aqueous extract of *Suoyang*
MSCs: mesenchymal stem cells
MTT: [3-(4,5-dimethylthiazol-2-yl)-2,5-diphenyltetrazolium bromide]
PTIO•: (2-phenyl-4,4,5,5-tetramethylimidazoline-3-oxide-1-oxyl)
ROS: reactive oxygen species
SD: standard deviation
TCM: tradition Chinese medicine
TPTZ: 2,4,6-tris(2-pyridyl-s-triazine)
Trolox: [(±)-6-hydroxyl-2,5,7,8-tetramethlychromane-2-carboxylic acid]
