# Supplementary figures and images for: Two phenolic antioxidants in Suoyang enhance viability of •OH-damaged mesenchymal stem cells: comparison and mechanistic chemistry
Source: Chem Cent J. 2017 Aug 25;11:84. doi: 10.1186/s13065-017-0313-1 (PMC5572787; doi:10.1186/s13065-017-0313-1)

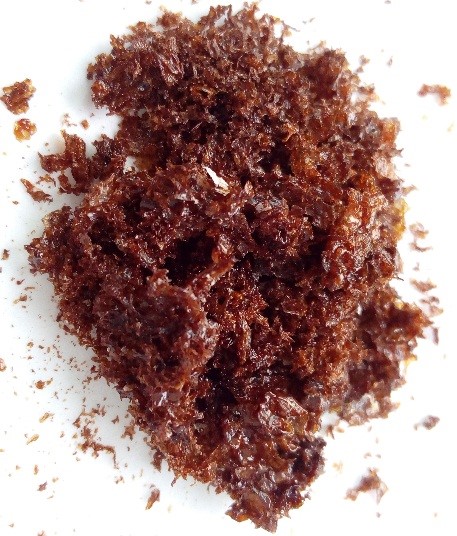

Supplement: Supplementary file 1 — Additional file 1. The appearance of the lyophilized aqueous extract of Suoyang. [file 13065_2017_313_MOESM1_ESM.jpg]

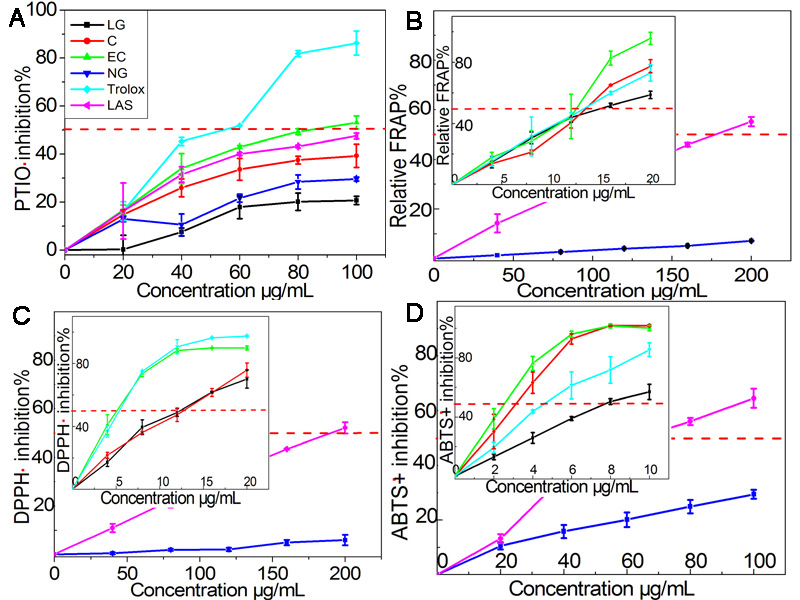

Supplement: Supplementary file 2 — Additional file 2. The dose response curves of PTIO, FRAP, DPPH, and ABTS assays. [file 13065_2017_313_MOESM2_ESM.tif]
